# Characterization and Genome Analysis of a Nicotine and Nicotinic Acid-Degrading Strain *Pseudomonas putida* JQ581 Isolated from Marine

**DOI:** 10.3390/md15060156

**Published:** 2017-05-31

**Authors:** Aiwen Li, Jiguo Qiu, Dongzhi Chen, Jiexu Ye, Yuhong Wang, Lu Tong, Jiandong Jiang, Jianmeng Chen

**Affiliations:** 1 College of Environment, Zhejiang University of Technology, Hangzhou 310014, China; liaiwen@zjut.edu.cn (A.L.); qiujiguo@njau.edu.cn (J.Q.); cdz@zjut.edu.cn (D.C.); yejiexu@zjut.edu.cn (J.Y.); 2 College of Life Sciences, Nanjing Agricultural University, Nanjing 210095, China; 3 Nanjing Yuanheng Institute for Environmental Studies Co., Ltd., Nanjing 210049, China; wangyuhong@yuanhenghj.com (Y.W.); Tonglu@yuanhenghj.com (L.T.)

**Keywords:** nicotine, nicotinic acid, biodegradation, genome, *Pseudomonas putida*, marine-derived bacterium

## Abstract

The presence of nicotine and nicotinic acid (NA) in the marine environment has caused great harm to human health and the natural environment. Therefore, there is an urgent need to use efficient and economical methods to remove such pollutants from the environment. In this study, a nicotine and NA-degrading bacterium—strain JQ581—was isolated from sediment from the East China Sea and identified as a member of *Pseudomonas putida* based on morphology, physio-biochemical characteristics, and 16S rDNA gene analysis. The relationship between growth and nicotine/NA degradation suggested that strain JQ581 was a good candidate for applications in the bioaugmentation treatment of nicotine/NA contamination. The degradation intermediates of nicotine are pseudooxynicotine (PN) and 3-succinoyl-pyridine (SP) based on UV, high performance liquid chromatography, and liquid chromatography-mass spectrometry analyses. However, 6-hydroxy-3-succinoyl-pyridine (HSP) was not detected. NA degradation intermediates were identified as 6-hydroxynicotinic acid (6HNA). The whole genome of strain JQ581 was sequenced and analyzed. Genome sequence analysis revealed that strain JQ581 contained the gene clusters for nicotine and NA degradation. This is the first report where a marine-derived *Pseudomonas* strain had the ability to degrade nicotine and NA simultaneously.

## 1. Introduction

With the continuous development of the tobacco industry, pyridine alkaloids are widely distributed in the environment [1,2,3]. The processing of tobacco produces a large number of liquid and solid wastes, which contain high concentrations of nicotine and nicotinic acid (NA), and can break the balance of the ecosystem and harm organisms when such waste comes into contact with the environment [4].

Currently, various methods are available to detoxify the toxic and hazardous substances in the polluted environment [5]; however, both physical and chemical methods are too expensive and ineffective in waste disposal management. Biological methods are efficient as well as cost effective, and hence play an important role in the clean-up of toxic and hazardous waste in a contaminated environment [5]. Nicotine is an amine composed of pyridine and pyrrolidine rings, and is a highly addictive drug that meets the same dependence criteria as cocaine, heroin, and alcohol [6,7]. NA is a pyridine derivative which is necessary for all living organisms [8,9]. It is involved in organic redox, promoting metabolism, and can be used to treat dermatitis and neurological disorders; nevertheless, a high concentration of NA in the human body and a living environment can cause a certain degree of harm. 

**Figure 1:**
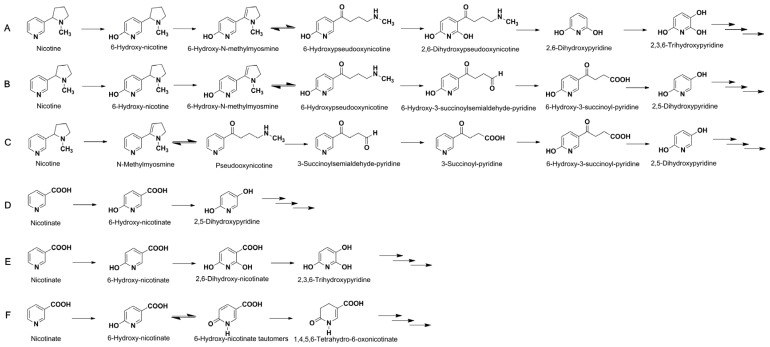
The overview of biodegradation pathways of (**A**–**C**) nicotine and (**D**–**F**) nicotinic acid (NA). (**A**) The pyrrolidine pathway; (**B**) the pyridine pathway; and (**C**) a variant of the pyridine and pyrrolidine pathways of nicotine degradation. (**D**) NA degradation pathways by *Pseudomonas*; (**E**) *Bacillus*; and (**F**) *Eubacterium* strains. The three consecutive arrows indicate the ring-cleaving which finally reaches the tricarboxylic acid cycle.

Nicotine is degraded by several microorganisms, such as *Arthrobacter nicotinovorans* [10], *Pseudomonas* [7,11], and *Shinella* strains [12,13,14,15,16]. These microorganisms degrade nicotine via different pathways, such as the pyrrolidine pathway (starting with the dehydrogenation of the pyrrolidine ring), the pyridine pathway (starting with the hydroxylation of the pyridine ring), and a variant of the pyridine and pyrrolidine pathways (Figure 1A–C), which have been well summarized in four recent reviews [5,10,11,17]. NA can degraded by *Bacillus* [18], *Comamonas* [19], *Eubacterium* [8], and *Pseudomonas* strains [9], and the main intermediate is 6-hydroxynicotinic acid (6HNA) (Figure 1D–F). The above-mentioned microorganisms only have the ability to degrade either nicotine or NA; thus far, no strains have been reported which are capable of degrading both simultaneously.

In our study, a new *Pseudomonas putida* strain JQ581 was isolated from sediment from the East China Sea and characterized. This strain could utilize both nicotine or NA as a sole carbon source. The genome of strain JQ581 was sequenced and analyzed.

## 2. Results and Discussion

### 2.1. Isolation and Identification of Strain JQ581

**Figure 2:**
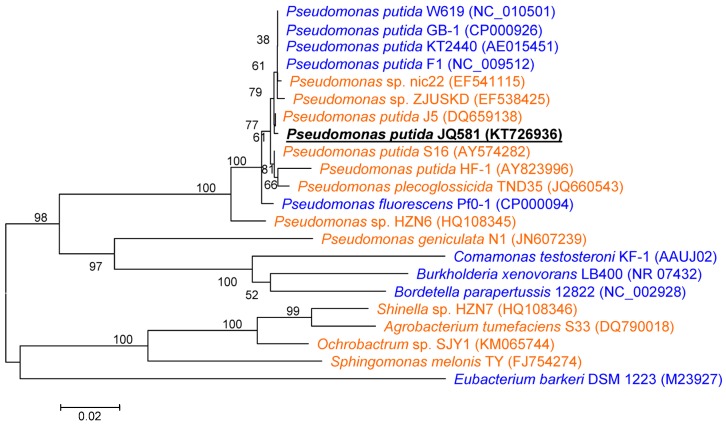
Phylogenetic analysis of strain JQ581 and related species by the neighbor-joining approach. The GenBank accession numbers are shown in parentheses. The scale bars represent 0.02 substitutions per site. The strains that could degrade nicotine are shown in orange: *Pseudomonas putida* S16 [7,20,21,22], *Pseudomonas* sp. nic22 [23], *Pseudomonas* sp. ZJUSKD [24], *Pseudomonas putida* J5 [25], *Pseudomonas putida* HF-1 [26], *Pseudomonas plecoglossicida* TND35 [27], *Pseudomonas* sp. HZN6 [28,29,30], *Pseudomonas geniculata* N1 [31], *Shinella* sp. HZN7 [16], *Agrobacterium tumefaciens* S33 [32], *Ochrobactrum* sp. SJY1 [33], *Sphingomonas melonis* TY [34]. The strains that could degrade NA are shown in blue: *Pseudomonas putida* KT2440, W619, GB-1, F1, and *Pseudomonas fluorescens* Pf0-1 [9], *Comamonas testosteroni* KF-1 [9], *Burkholderia xenovorans* LB400 [9], *Bordetella parapertussis* 12822 [9], and *Eubacterium barkeri* DSM 1223 [8].

In this study, more than 30 bacterial strains able to utilize nicotine as the sole carbon source for their growth were isolated from sediment from the East China Sea; however, only one of them—JQ581—could also degrade NA for cell growth. When strain JQ581 was cultured with nicotine, a bright yellowish green color was found. When strain JQ581 was cultured with NA, the media remained colorless throughout the degradation progress. Strain JQ581 is a Gram-negative, aerobic, rod-shaped bacterium with spores of 1.0–1.5 μm in length and 0.6–0.9 μm in width. Colonies grown on Luria–Bertani (LB) agar plates were beige, smooth, and opaque, with a diameter of 2–3 mm. Strain JQ581 could utilize glucose, glycerol, and citric acid for cell growth, but not maltose or sucrose. Detailed physio-biochemical characteristics of strain JQ581 were analyzed by the BIOLOG system, and the results indicated that JQ581 was a member of *Pseudomonas putida* (Table 1). Strain JQ581 could grow at 1% NaCl, but not at 4% or 8%. JQ581 is resistant to ampicillin, chloramphenicol, streptomycin, rifamycin, troleandomycin, and lincomycin, but susceptible to gentamicin, kanamycin, and minocycline. A phylogenetic tree was constructed based on 16S rRNA sequences (Figure 2). Analysis of the 16S rDNA gene sequences demonstrated that strain JQ581 was grouped among the *Pseudomonas putida* species. In conclusion, strain JQ581 was identified as *Pseudomonas putida* based on morphology, physio-biochemical characteristics, and 16S rDNA gene analysis.

### 2.2. Utilization of Nicotine/NA for Growth by Strain JQ581

**Figure 3:**
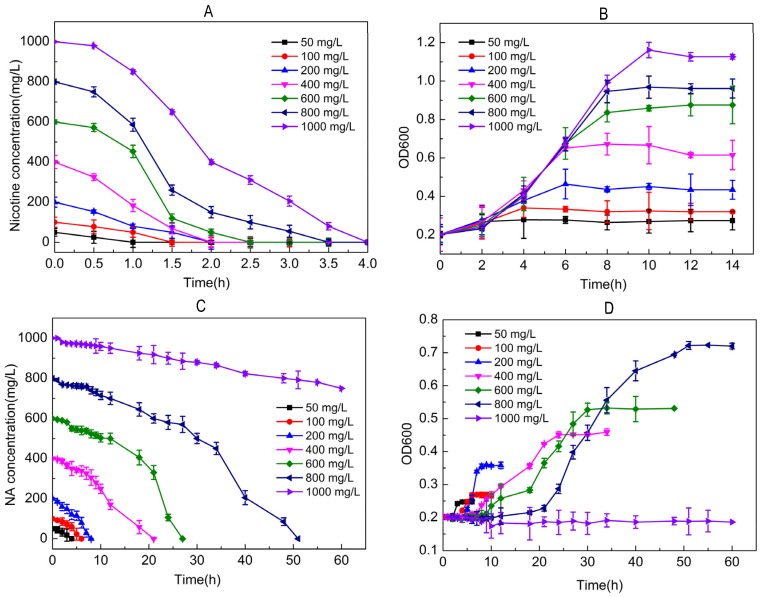
(**A**,**B**) Biodegradation of nicotine and cell growth of strain JQ581; (**C**,**D**) Biodegradation of NA and cell growth of strain JQ581.

The relationship between nicotine degradation and cell growth of JQ581 is shown in Figure 3. The initial concentrations of nicotine were 50–1000 mg/L, and the initial inoculum concentration was OD600 0.2. When nicotine concentration was lower than 600 mg/L, the substrate completely degraded in 2 h (Figure 3A). When the nicotine concentration was up to 1000 mg/L, it degraded within 4 h. The cell growth of strain JQ581 is shown in Figure 3B. The OD600 of strain JQ581 increased from 0.2 to 1.15 ± 0.11. When the NA was degraded by strain JQ581 with different initial concentrations of 50–1000 mg/L, degradation was much slower than that of nicotine (Figure 3C). The 400 and 800 mg/L nicotine samples degraded completely after 24 h and 48 h, respectively. The OD600 of strain JQ581 only increased from 0.2 to 0.67 ± 0.05. When the NA concentration reached 1000 mg/L, only one third of NA reduced after incubation for 72 h. The OD600 values did not change significantly, which might be due to the toxic effect of NA on JQ581. The above-mentioned properties make strain JQ581 a good candidate for applications in the bioaugmentation treatment of nicotine/NA-contaminated water and soil. 

### 2.3. Metabolic Pathway of Nicotine and NA Biodegradation

**Figure 4:**
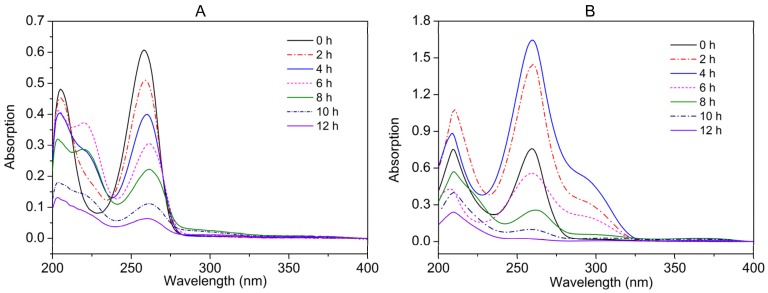
(**A**) Spectral changes during nicotine; and (**B**) NA degradation by strain JQ581.

**Figure 5:**
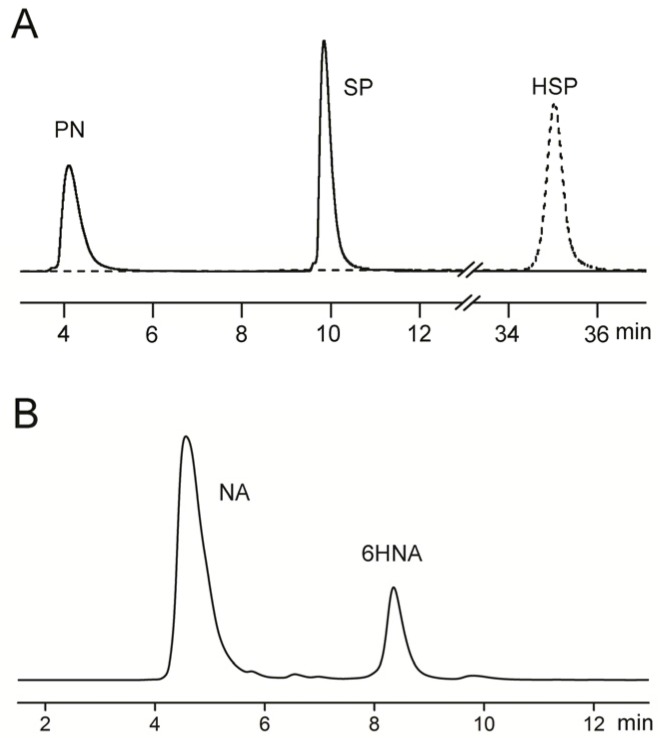
HPLC profile of (**A**) nicotine; and (**B**) NA degradation by strain JQ581; (**A**) The sample collected at 6 h is shown by the solid line. 6-Hydroxy-3-succinoyl-pyridine (HSP) standard is shown as the dotted line; (**B**) The sample was collected at 6 h. 6HNA: 6-hydroxynicotinic acid; PN: pseudooxynicotine; SP: 3-succinoyl-pyridine.

The nicotine degradation intermediates were identified by UV, high performance liquid chromatography (HPLC), and liquid chromatography-mass spectrometry (LC-MS) analyses. The altered UV absorption during nicotine degradation indicated the presence of metabolites (Figure 4A). The wavelength of maximum UV absorption (λ_max_) of nicotine at 259 nm changed to 223 nm and 263 nm, before finally disappearing. HPLC results showed that two major peaks appeared, and retention times were identical to that of pseudooxynicotine (PN) (4.19 min) and 3-succinoyl-pyridine (SP) (10.06 min), respectively (Figure 5A). LC-MS results showed that the molecular ion peaks ([M + H]^+^) were 179.1 and 180.1, which were identical to standard PN and SP, respectively. However, no peaks of 6-hydroxy-3-succinoyl-pyridine (HSP) were found. 

The NA degradation intermediates were identified by UV and high performance liquid chromatogram analyses. The altered UV absorption during nicotine degradation is shown in Figure 4B. The λ_max_ of NA was determined at 259 nm. The UV absorption at 259 nm and 295 nm increased at first, and then finally disappeared. HPLC results showed that one peak appeared, and the retention time was 8.53 min (Figure 5B)—identical to the standard 6HNA. LC-MS results showed that the molecular ion peak ([M + H]^+^) was 165.1, which was identical to 6HNA. 

### 2.4. Genome Features of Strain JQ581

The draft genome of strain JQ581 was 6,563,906 bp. The genome size (6.56 Mb) was slightly larger than the reported *Pseudomonas putida* genomes (an average of 6.08 Mb) [35]. It contained 93 contigs, with an average contig length of 49.93 kb, a median coverage depth of 75-fold, and an average GC content of 63.00%. The genome prediction and annotation was performed with Rapid Annotation using Subsystems Technology (RAST) and Prokaryotic Genome Annotation Pipeline (PGAP). A total of 5723 coding sequences, 72 tRNA sequences, 4 ncRNA genes, and 1 rRNA sequence were identified. 

### 2.5. Genome Sequence Analysis and the Identification of Gene Clusters for Nicotine and NA Degradation

**Figure 6:**
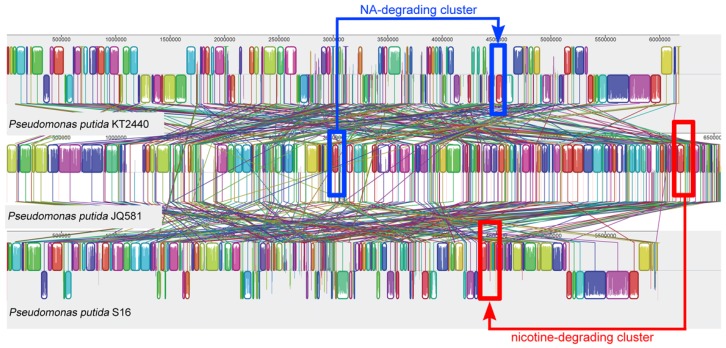
Alignment of the genomes from strain KT2440, JQ581, and S16 using the MAUVE software. Identically-colored boxes indicate homologous regions in the three sequences. The gene clusters for biodegradation of nicotine and NA are shown in red and blue, respectively.

**Figure 7:**
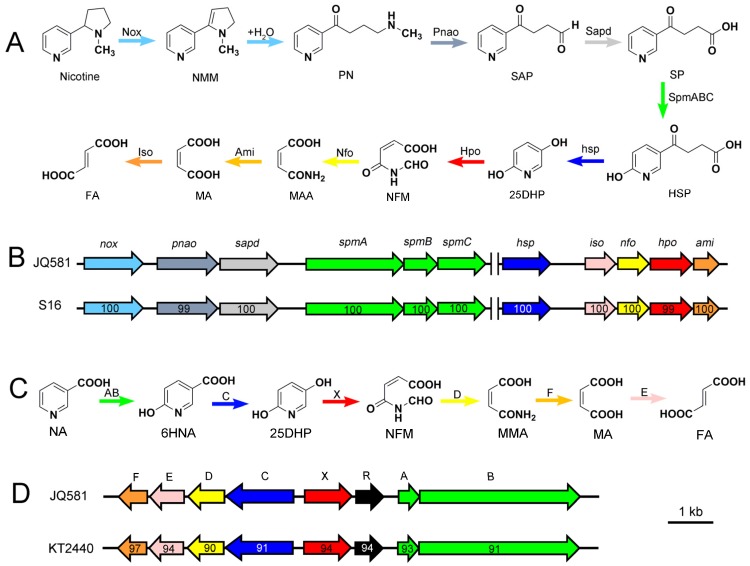
(**A**) Proposed nicotine degrading pathway by strain JQ581; (**B**) Genetic organization of the nicotine cluster in strains JQ581 and S16 [7]; (**C**) Proposed NA degrading pathway by strain JQ581; (**D**) Genetic organization of the NA cluster in strains JQ581 and KT2440 [9]; The arrows in (C) and (D) indicate the size and direction of each gene. NMM: N-Methylmyosmine; SAP: 3-Succinoylsemialdehyde-pyridine; FA: Fumaric acid; MA: Maleic acid; MAA: Maleamic acid; 25DHP: 2,5-Dihydroxypyridine. Numbers within the arrows indicate the DNA sequence identity with the orthologous gene from strain JQ581.

Nicotine and NA could be degraded by *Pseudomonas putida* strains S16 [7] and KT2440 [9], respectively. Therefore, the genes responsible for the substrate degradation in strain JQ581 were compared with these two strains. The genome sequences of strains JQ581, S16, and KT2440 were aligned using the MAUVE v.2.3.1 software (Figure 6). The symmetric identities of strain JQ581 with S16 and KT2440 were 66.71% and 66.59%, respectively. As expected, the nicotine degradation gene cluster exists in strain JQ581 and S16, while the NA degradation gene cluster exists in strain JQ581 and KT2440. As shown in Figure 7A,B, the nicotine degradation gene cluster was found and was identical to that of strain S16 (location at 4,580,001–4,625,700 bp). The DNA sequence identities of these genes appreciated 100%. Furthermore, an NA degradation gene cluster of strain JQ581 was found to be identical to that of strain KT2440 (location at 4,441,001–4,454,000 bp) (Figure 7C,D). The identities of these genes were approximately 91–97%. The high DNA sequence similarities of the NA degradation cluster between strain JQ581 and KT2440 indicate that they degrade NA via the same pathway. A similar deduction also applied to nicotine degradation between strain JQ581 and S16, although the intermediates HSP and 2,5-Dihydroxypyridine(25DHP) were not detected in the supernatant during nicotine degradation by strain JQ581. To date, a large number of nicotine or NA-degrading strains have been isolated and identified (Figure 2). However, to the best of our knowledge, strain JQ581 was the first identified bacterium to be capable of degrading nicotine and NA simultaneously.

## 3. Materials and Methods

### 3.1. Chemicals and Media

Nicotine (>99%) was purchased from Sigma-Aldrich (St. Louis, MS, USA). Pseudooxynicotine (PN) (98%) and 3-succinoyl-pyridine (SP) (98%) were purchased from Toronto Research Chemicals, Inc. (Toronto, ON, Canada). The 6-hydroxy-3-succinoyl-pyridine (HSP) standard was prepared as per Reference [12]. Nicotinic acid (NA) (>99%) and 6-hydroxynicotinic acid (6HNA) (>98%) were obtained from J&K Scientific Ltd., (Shanghai, China). Chromatography-grade methanol was purchased from J&K Scientific Ltd., China. All other chemicals and solvents used in this experiment were commercially available. The components of mineral salts medium (MSM) and Luria–Bertani (LB) medium were the same as in our previous report [28]. Both media were adjusted to pH 7.0 and autoclaved to sterilize at 121 °C for 25 min. Solid medium was prepared by adding 2.0% agar (*w/v*).

### 3.2. Screening and Isolation of Bacteria of Degrading Nicotine and NA

Sediment samples were collected from the seaside by the East China Sea. The samples were incubated with LB liquid medium for 3 days to sufficiently assure the viability of most of the cells in the sample. Thereafter, all cultured bacterial cells were collected by centrifugation at low speed and washed twice with sterile water. The samples were incubated under aerobic conditions at 30 °C. At 7 days post-incubation, portions (5%, *v/v*) were transferred into another fresh MSM containing nicotine (500 mg/L) and incubated for another 7 days. After several serial transfers, samples were spread on MSM agar plates containing nicotine (500 mg/L). The well-isolated colonies were transferred into 250 mL Erlenmeyer flasks containing 50 mL sterilized MSM and incubated at 30 °C on a rotary shaker at 180 rpm. Individual colonies were further transferred into MSM to confirm the ability to degrade nicotine (500 mg/L) and NA (500 mg/L). One strain (JQ581) showed high degrading efficiency of both nicotine and NA, and was selected for further studies.

### 3.3. Identification of Strain that Degrades Nicotine and NA

The selected bacterium JQ581 was identified by morphological, physico-biochemical characterization, and the 16S rRNA gene sequence. Genomic DNA was extracted using a Bacterial Genomic DNA Mini Kit (Sangon, Shanghai, China). The 16S rRNA gene was amplified by PCR using the universal primers 27F and 1492R [36]. The PCR products were cloned into the vector pMD18-T (TaKaRa Biotechnology Co. Ltd., Dalian, China) and transformed into *Escherichia coli* DH5α cells. The PCR product was sequenced by Sangon Biotechnology Co. Ltd. (Shanghai, China), and the obtained sequence was compared to sequences in the GenBank database using the BLAST program. The 16S rRNA gene sequence of strain JQ581 and related sequences retrieved from GenBank were aligned using Clustal W. The phylogenetic tree was analyzed and constructed with the neighbor-joining method by MEGA version 5.0 [37].

### 3.4. Biodegradation Assay

The degradation of nicotine/NA by strain JQ581 was performed in 250 mL Erlenmeyer flasks with 100 mL of sterile MSM. Inocula were prepared by inoculating the individual colony into 100 mL of LB at 30 °C with shaking at 180 rpm. The bacterial cells in the late-exponential growth phase were harvested by centrifugation at 6000× *g* for 5 min and washed with MSM and resuspended in MSM. Strain JQ581 was pre-cultured in MSM with 100 mg/L nicotine or NA for 12 h. Next, the strain was harvested and the OD600 was adjusted to 1.0. Five percent of this suspension was used as the inocula for subsequent studies. Different initial nicotine/NA concentrations were set as 50, 100, 200, 400, 600, 800, and 1000 mg L^−1^. The experiment was conducted in triplicate at 30 °C on a rotary shaker at 150 rpm. The non-inoculated samples served as controls. Samples were collected periodically from the cultures. Growth was monitored by measuring the optical density (OD) value at 600 nm, and the residual nicotine/NA concentration was measured by HPLC.

### 3.5. Identification of Nicotine/NA Biodegradation Metabolites

To identify nicotine/NA and their metabolites during biodegradation, bacterial strains were grown in MSM containing nicotine (400 mg/L) or NA (200 mg/L). Non-inoculated samples containing the same amount of nicotine/NA were used as control. Samples were collected at appropriate intervals. The supernatant from the samples was obtained by centrifugation at 12,000 rpm at 4 °C for 10 min. After filtration with 0.22 μm membrane (Millipore, Billerica, MA, USA), the samples were subjected to UV or HPLC analysis.

### 3.6. Analytical Methods

The presence of nicotine/NA in the supernatants with 0.1 M HCl was primarily monitored by UV/Vis Spectrophotometer (JASCO V-550, Japan). Spectral data were collected at wavelengths from 200 nm to 400 nm. 

The nicotine/NA residues in the cultures were analyzed on an Agilent 1200 HPLC system equipped with a C18 reversed phase column (250 × 4.60 mm, 5 μm) at 260 nm based on retention time and peak area of the pure standard. A mixture of methanol:water:formic acid (100:900:2, *v/v/v*) was used as the mobile phase at a flow rate of 0.6 mL min^−1^, and the column temperature was 30 °C.

### 3.7. Draft Genome Sequencing and Bioinformatic Analysis of Strain JQ581

Total DNA of strain JQ581 was extracted using a QIAGEN DNA kit. Library preparation and genome sequencing were performed by BaseClear BV (Leiden, The Netherlands). A paired-end DNA library with a mean gap length size between 200 and 350 bp was sequenced, with average reads of 250 bp, on an Illumina Miseq2000 apparatus. The draft genome sequence was assembled using the SOAPdenovo version 2.04. Functional annotation was performed with the Rapid Annotation Subsystem Technology (RAST) server [38] and the National Center for Biotechnology Information’s Prokaryotic Genome Automatic Annotation Pipeline (PGAAP). The reported genes involving the degradation of nicotine or NA were used to blast the genome of strain JQ581 in NCBI. 

### 3.8. Deposition of the Nucleotide Sequence Accession Number

Strain JQ581 has also been deposited in the China Centre for Type Culture Collection under accession number CCTCC AB 2016095. The nucleotide sequences of the 16S rDNA sequence and the draft genome sequence of strain JQ581 have been deposited in GenBank under the accession numbers KT726936 and LWDW00000000, respectively.

## 4. Conclusions

A *Pseudomonas putida* strain JQ581 which capable of degrading nicotine and NA simultaneously was isolated and identified from marine. The strain could degrade 1000 mg/L nicotine and 800 mg/L NA efficiently. The degradation intermediates of nicotine are PN and SP. NA degradation intermediates were identified as 6HNA. The draft genome of strain JQ581 was sequenced and analyzed. Genome sequence analysis revealed that strain JQ581 contained the gene clusters for nicotine and NA degradation. This is study provided the first marine-derived *Pseudomonas* strain degrading nicotine and NA simultaneously.

## Figures and Tables

**Table 1:** The biochemical phenotypes of strain JQ135 on the BIOLOG system.

Tests	Result	Tests	Result
1 Negative Control	−	49 Methyl Pyruvate	w
2 Dextrin	−	50 d-Lactic Acid Methyl Ester	−
3 d-Maltose	−	51 α-Keto-Glutaric Acid	w
4 d-Trehalose	−	52 Nalidixic Acid	+
5 d-Cellobiose	−	53 Lithium Chloride	w
6 Gentiobiose	−	54 Potassium Tellurite	+
7 Sucrose	−	55 Tween 40	−
8 d-Turanose	−	56 γ-Amino-Butryric Acid	+
9 Stachyose	−	57 α-Hydroxy-Butyric Acid	−
10 d-Raffinose	−	58 β-Hydroxy-d,l-Butyric Acid	−
11 α-d-Lactose	−	59 α-Keto-Butyric Acid	−
12 d-Melibiose	−	60 Acetoacetic Acid	w
13 β-Methyl-d-Glucoside	−	61 Gelatin	−
14 d-Salicin	−	62 Glycyl-l-Prolin	−
15 *N*-Acetyl-d-Glucosamine	−	63 l-Alanine	+
16 *N*-Acetyl-β-DMannosamine	−	64 l-Arginine	+
17 *N*-Acetyl-d-Galactosamine	−	65 l-Aspartic Acid	+
18 *N*-AcetylNeuraminic Acid	−	66 l-Glutamic	+
19 α-d-Glucose	w	67 l-Histidine	+
20 d-Mannose	w	68 l-Pyroglutamic	+
21 d-Fructose	w	69 l-Serine	w
22 d-Galactose	w	70 Lincomycin	+
23 3-Methyl Glucose	−	71 Guanidine HCl	w
24 d-Fucose	w	72 Niaproof 4	+
25 l-Fucose	w	73 Pectin	−
26 l-Rhamnose	−	74 d-Galacturonic Acid	+
27 Inosine	−	75 l-Galactonic Acid Lactone	+
28 1% Sodium Lactate	+	76 d-Gluconic Acid	+
29 Fusidic Acid	+	77 d-Glucuronic Acid	+
30 d-Serine	+	78 Glucuronamide	+
31 d-Sorbitol	−	79 Mucic Acid	+
32 d-Mannitol	−	80 Quinic Acid	+
33 d-Arabitol	−	81 d-Saccharic Acid	+
34 myo-Inositol	−	82 Vancomycin	+
35 Glycerol	w	83 Tetrazolium Violet	+
36 d-Glucose-6-PO4	−	84 Tetrazolium Blue	+
37 d-Fructose-6-PO4	+	85 l-Lactic Acid	w
38 d-Aspartic Acid	+	86 Citric Acid	+
39 d-Serine	+	87 d-Malic Acid	+
40 Troleandomycin	+	88 l-Malic Acid	+
41 Rifamycin SV	+	89 Bromo-Succinic Acid	w
42 Minocycline	−	90 Formic Acid	−
43 Positive Control	+	91 Propionic Acid	w
44 pH 6	+	92 Acetic Acid	w
45 pH 5	+	93 Aztreonam	w
46 1% NaCl	+	94 Sodium Butyrate	−
47 4% NaCl	−	95 Sodium Bromate	−
48 7% NaCl	−	96 p-Hydroxy-Phenylacetic Acid	w

Notes: − = negative reaction; + = positive reaction; w = weak positive reaction.

## Supplementary Materials

The following are available online at http://www.mdpi.com/1660-3397/15/6/156/s1. Figure S1: The HPLC profile of standard product of nicotine (A) and its standard curve (B), Figure S2: The HPLC profile of standard product of nicotinic acid (A) and its standard curve (B), Figure S3: The HPLC profile of standard products of PN, SP and HSP, Figure S4: The HPLC profile of standard products of NA and 6HNA.
