# Supplementary material for: Characterization and Genome Analysis of a Nicotine and Nicotinic Acid-Degrading Strain Pseudomonas putida JQ581 Isolated from Marine
Source: Mar Drugs. 2017 May 31;15(6):156. doi: 10.3390/md15060156 (PMC5484106; doi:10.3390/md15060156)
Supplement: Supplementary file 1 [file marinedrugs-15-00156-s001.pdf]

marine drugs

Supporting Information

Characterization and genome analysis of a nicotine and nicotinic acid–degradingstrain *Pseudomonas putida* JQ581 isolated from marine

Aiwen Li[1,†], Jiguo Qiu[1,2,†], Dongzhi Chen[1], Jiexu Ye[1], Yuhong Wang[3], Lu Tong[3], Jiandong Jiang[2,*], and Jianmeng Chen[1,*]

[1] College of Environment, Zhejiang University of Technology, Hangzhou, 310014, China; liaiwen@zjut.edu.cn (A.L.); cdz@zjut.edu.cn (D.C.); yejiexu@zjut.edu.cn (J.Y.)
[2] College of Life Sciences, Nanjing Agricultural University, Nanjing, 210095, China; qiujiguo@njau.edu.cn (J.Q.)
[3] Nanjing Yuanheng Institute for Environmental Studies Co., Ltd., Nanjing, 210049, China; wangyuhong@yuanhenghj.com (Y.W.); Tonglu@yuanhenghj.com (L.T.)

* Correspondence:jchen@zjut.edu.cn (J.C.), Tel.: +86-571-88320386, Fax: +86-571-88320882; or jiang_jjd@njau.edu.cn (J.J.), Tel.: +86-25-84399726, Fax: +86-25-84396314
† These authors contributed equally to this work.

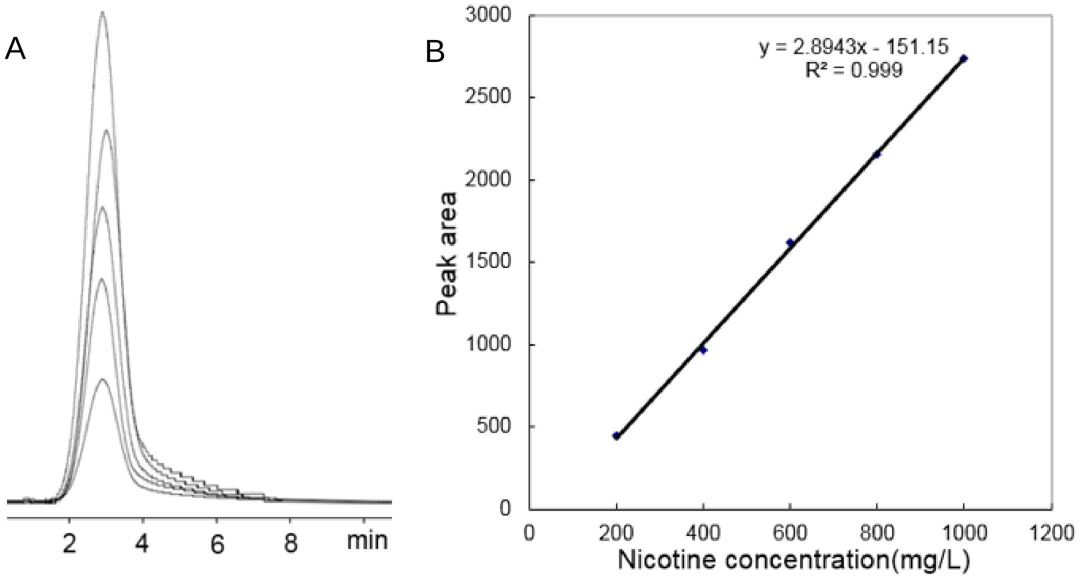

Figure S1. The HPLC profile of standard product of nicotine (A) and its standard curve (B).

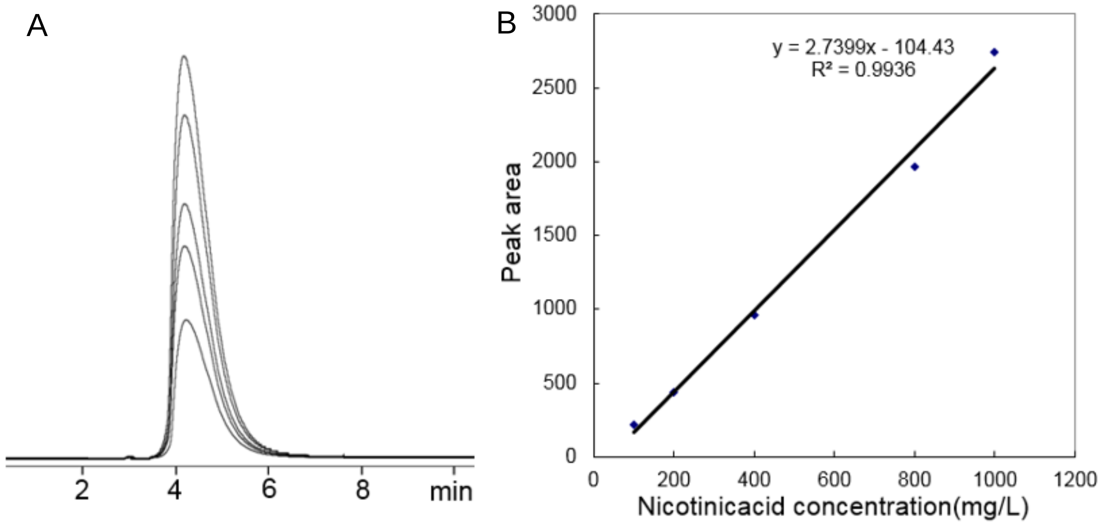

Figure S2. The HPLC profile of standard product of nicotinic acid (A) and its standard curve (B).

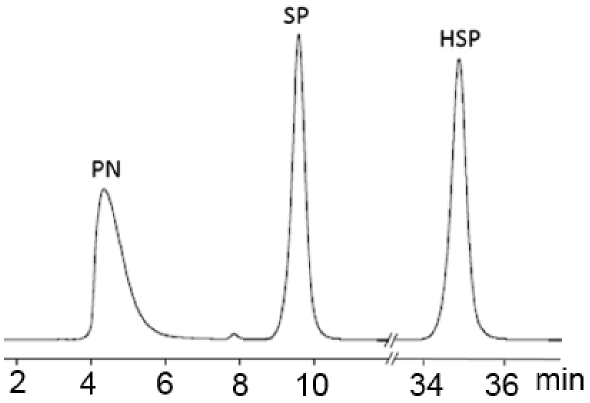

Figure S3. The HPLC profile of standard products of PN, SP and HSP.

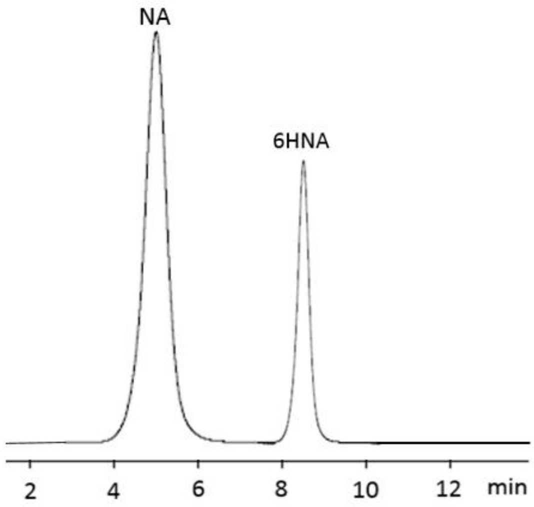

Figure S4. The HPLC profile of standard products of NA and 6HNA.
